# Different Chemotherapy Regimens and Pathologic Complete Response in Triple-Negative Breast Cancer: An Updated Network Meta-Analysis of Phase 3 Trials

**DOI:** 10.3390/medicina60020341

**Published:** 2024-02-19

**Authors:** Fausto Petrelli, Gianluca Tomasello, Maria Chiara Parati, Antonio Ghidini, Michele Ghidini, Karen Borgonovo, Mary Cabiddu, Mara Ghilardi, Roberto Reduzzi, Donatella Gambini, Alberto Zaniboni, Giovanni Faustinelli, Ornella Garrone

**Affiliations:** 1 Oncology Unit, ASST Bergamo Ovest, 24047 Treviglio, Italy; faupe@libero.it (F.P.); mchiara.parati@gmail.com (M.C.P.); karen_borgonovo@asst-bgovest.it (K.B.);; 2 Medical Oncology Unit, Fondazione IRCCS Ca’ Granda Ospedale Maggiore Policlinico, 20122 Milan, Italyornella.garrone@policlinico.mi.it (O.G.); 3 Oncology Unit, Casa di Cura Igea, 20129 Milano, Italy; antonioghidini@hotmail.com; 4 ASP IMMEeS & PAT, 20146 Milano, Italy; mary_cabiddu@yahoo.it (M.C.); giovanni_faustinelli@asst-bgovest.it (G.F.); 5 Breast Unit, ASST Bergamo Ovest, 24047 Treviglio, Italy; roberto_reduzzi@asst-bgovest.it; 6 Oncology Unit, Fondazione Poliambulanza, 25124 Brescia, Italy; alberto.zaniboni@poliambulanza.it

**Keywords:** breast cancer, triple negative, neoadjuvant, chemotherapy, meta-analysis, pathologic complete response

## Abstract

*Background and Objectives*: Currently, the standard treatment for non-metastatic triple-negative breast cancer (TNBC) consists of a systemic neoadjuvant (or perioperative) anthracycline plus taxane-based chemotherapy, delivered either sequentially or concomitantly. We performed a network meta-analysis (NMA) to compare the relative efficacy of different neoadjuvant treatments for TNBC in terms of pathologic complete response (pCR). *Materials and Methods*: The MEDLINE, Embase, and Cochrane databases were searched from database inception to 1 November 2023. Randomized clinical trials were used that enrolled adults with stage I-III TNBC and provided data on pCR defined as residual ypT0/TisN0M0. Between-group comparisons were estimated using risk ratios (RRs) with 95% credible intervals (95% CrIs). The primary outcome was the pCR rate. *Results*: 1129 citations were screened, and 12 randomized clinical trials were included. In Bayesian comparisons, all regimens, except anthracycline/taxanes plus gemcitabine or capecitabine, resulted in a higher pCR than the standard regimen in both direct and indirect comparisons. In particular, immunotherapy-based regimens resulted in more than double the pCR compared to historical regimens (RR = 2.3, 95% CI 1.9–2.9) and ranked as being the optimal regimen with a probability of 97%. Disease-free survival was better for immune checkpoint inhibitor-based chemotherapy (HR = 0.36, 95% 1.21–2.09) than for historical regimens. *Conclusion*: This meta-analysis confirmed that incorporating immunotherapy with neoadjuvant platinum-based chemotherapy is the best option to guarantee remarkable pathologic downstaging and improve clinical outcomes.

## 1. Introduction

Neoadjuvant chemotherapy, a treatment approach administered before primary surgery, has revolutionized the management of breast cancer (BC), particularly in aggressive subtypes, such as triple-negative breast cancer (TNBC). Its primary goal is to shrink tumors, making them more amenable to conservative surgical procedures and reducing the presence of early micrometastases [1]. While this strategy does not directly improve overall survival, it plays a crucial role in patient management and prognosis.

The incorporation of taxanes into anthracycline-based regimens has been a significant advancement in this approach. Regardless of the order of administration, this combination has demonstrated improved response rates, which is a critical factor in determining the subsequent course of treatment [2]. The achievement of a pathologic complete response (pCR), particularly defined as ypT0pTispN0 stage post-surgery, is a key marker of effective treatment, correlating with improved long-term outcomes in aggressive BC subtypes. While chemotherapy may yield initial effectiveness, it is frequently accompanied by resistance, recurrence, and significant toxicity. Moreover, those who develop metastatic cancer typically have survival rates on the order of months. Given this reasoning, there is a pressing requirement for drugs that are both more efficient and less harmful.

In the specific context of non-metastatic TNBC, a particularly challenging subtype due to the lack of hormonal and HER2-targeted therapies, standard treatment usually consists of systemic neoadjuvant chemotherapy with anthracycline and taxane. Recent small-scale phase 2 studies have suggested that the addition of platinum-based drugs such as carboplatin or cisplatin could further enhance treatment efficacy. These studies indicate that such additions can lead to higher rates of pCR, an essential predictor of long-term disease control and survival [3,4]. The introduction of immune checkpoint inhibitors such as pembrolizumab into the standard neoadjuvant chemotherapy regimen has marked another significant milestone in TNBC treatment [5]. These agents function by unleashing the immune system against cancer cells, and their integration into treatment protocols has led to a substantial increase in the overall pCR rate. This improvement in the response rate is noteworthy, as it occurs independently of PD-L1 expression, a biomarker previously thought to predict the benefit of immunotherapy. Furthermore, pembrolizumab has shown a remarkable improvement in 3-year event-free survival rates, significantly reducing the risk of cancer recurrence or death.

Network meta-analysis (NMA) has emerged as a vital tool for evaluating the relative effectiveness of diverse and complex treatment regimens. Given the scarcity of direct head-to-head comparisons between traditional and newer TNBC treatments, NMA offers a more comprehensive approach by integrating both direct and indirect evidence from various randomized controlled trials (RCTs). This method allows for a more nuanced understanding of how different neoadjuvant therapies compare in terms of disease-free survival and pCR rates.

The current study’s NMA was particularly focused on analyzing large phase III trials, aiming to provide an updated picture of the most effective neoadjuvant therapy options for patients with localized or locally advanced TNBC. By synthesizing data from these trials, the analysis sought to guide clinical decision making, offering insights into which combinations of drugs yield the best outcomes in terms of tumor response and long-term disease control.

## 2. Material and Methods

This study followed the PRISMA extension statement to report NMA. We systematically searched online databases, including MEDLINE, Embase, and the Cochrane Central Register of Controlled Trials, for all phase 3 trials published up to 1 November 2023. For search terms, we used the medical subject headings of ‘breast cancer’, ‘neoadjuvant chemotherapy’, ‘induction chemotherapy’, ‘primary chemotherapy’, ‘triple negative’, ‘HER-2 negative’, ‘ER-negative and PgR-negative’, and ‘pathologic complete response’. The inclusion criteria were as follows: (a) phase 3 trials; (b) patients with localized or locally advanced TNBC; (c) trials that compared standard anthracycline/taxane-based chemotherapy with any regimens containing anthracycline/taxanes-based plus or minus another agent or a more intensified schedule (e.g., dose-dense); (d) trials that reported pCR rates in the intention-to-treat population; and (e) articles published in English. We excluded the following: (a) randomized phase 2 trials, (b) trials that compared perioperative chemotherapy, (c) a former version of the same trial, and (d) studies with unavailable full text. The quality of the included studies was assessed using the revised Cochrane risk-of-bias tool for randomized trials (RoB2 tool) in two independent reviews (FP and GT).

The primary outcome was the pCR value, defined as the stage ypT0/ispN0 at surgery (amount of events/number of patients for each arm). The heterogeneity between the studies was assessed using the Q test and I^2^ statistics. The fixed-effects or random-effects model was chosen based on the I^2^ value (<50% or >50%, respectively). The secondary outcome was disease-free survival (DFS) or, where available, event-free survival (EFS).

Fixed-effects and consistency models were used in the NMA. Non-informative priors were set, and posterior distributions were obtained using 40,000 iterations after 20,000 burns and a thinning interval of 10. Network meta-analysis results for pCR are reported as relative risks (RRs) with 95% credible intervals (CrIs). The probability of each treatment regarding survival outcomes was ranked according to the RRs and posterior probabilities. Network meta-analysis results for EFS/DFS are reported as hazard ratios (HRs) with 95% credible intervals (CrIs). The probability of each treatment regarding survival outcomes was ranked according to the HRs and posterior probabilities. Statistical significance was set at *p* < 0.05, indicating statistical significance. For EFS/DFS, we calculated the mean log HR and its standard error and entered it into the model, while for pCR we entered the number of events in each arm.

The assumptions for network meta-analysis, i.e., ‘consistency’ for mixed treatment comparison and ‘transitivity’ for indirect comparison, will be examined before analysis. The inconsistency (i.e., the disparity between direct and indirect estimates) will be tested through the use of mixed comparison modeling using the inconsistency factor for each closed loop. The transitivity assumption will be qualitatively examined by visualizing the distribution of potential effect modifiers across the trials. Finally, we calculated the relative ranking of agents for each outcome as their surface under the cumulative ranking (SUCRA), which represents the percentage of efficacy or safety achieved by an agent compared with an imaginary agent that is always the best without uncertainty. A higher SUCRA score meant a higher ranking for efficacy outcomes. Network meta-analyses were performed under the Bayesian framework using the “gemtc” package (https://gemtc.drugis.org accessed on 21 December 2023) [6].

## 3. Results

**Figure 1:**
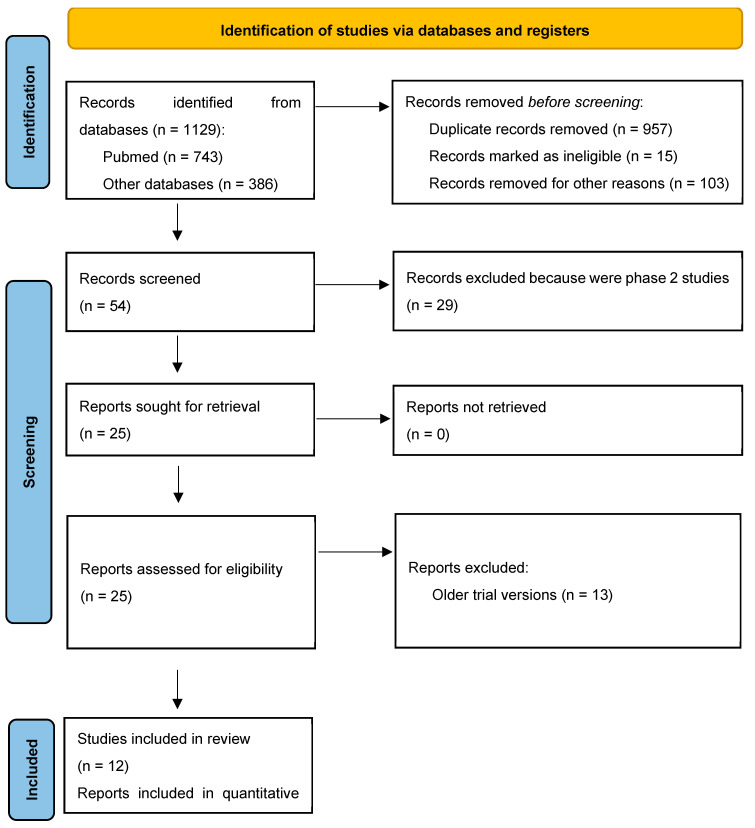
Flow diagram of the included studies.

The study selection process is outlined in Figure 1. Initially, 1129 items were identified through a literature search. After the screening process, 25 articles were shortlisted. Of these, 13 were excluded due to their focus on chemotherapies not involving anthracycline or taxane or their failure to provide pCR data specifically for TNBC patients. Consequently, 12 studies were deemed suitable and met the stringent inclusion criteria, as referenced in citations [5,7,8,9,10,11,12,13,14,15,16,17,18,19].

### 3.1. Study Characteristics

The 12 selected phase III studies offered n = 10 direct comparisons. Nine studies directly compared anthracycline/taxane regimens augmented with other agents (carboplatin plus or minus veliparib, capecitabine, gemcitabine, bevacizumab, vinorelbine/capecitabine or nab-paclitaxel). Two studies focused on immunotherapy-based regimens (atezolizumab and pembrolizumab). The Mittendorf et al. trial compared anthracyclines/taxanes-based chemotherapy with or without atezolizumab, and the Schmid et al. study added pembrolizumab to an anthracycline/carboplatin and taxanes-based regimen. One pivotal study compared a platinum-based regimen against a dose-dense schedule involving anthracycline/taxanes. Each study has, respectively, 3, 4 and 6 arms. In Loibl et al. PARP + platinum-based and platinum-based chemotherapy were compared for the pCR endpoint to the standard of care arm. In Earl et al., two different chemotherapy sequences were compared with the same two sequences with the addition of gemcitabine, and finally, in the study by Bear et al., three different anthracycline–taxanes-based chemotherapies were compared with the same regimens plus bevacizumab. In the last two studies, comparisons for pCR and/or DFS/EFS regarded the addition of gemcitabine and bevacizumab.

Table 1 provides an exhaustive listing of the baseline characteristics for all of the included studies. Remarkably, each study was assessed and found to possess a low risk of bias, ensuring the reliability and validity of their findings.

### 3.2. Pathologic Complete Response

**Figure 2:**
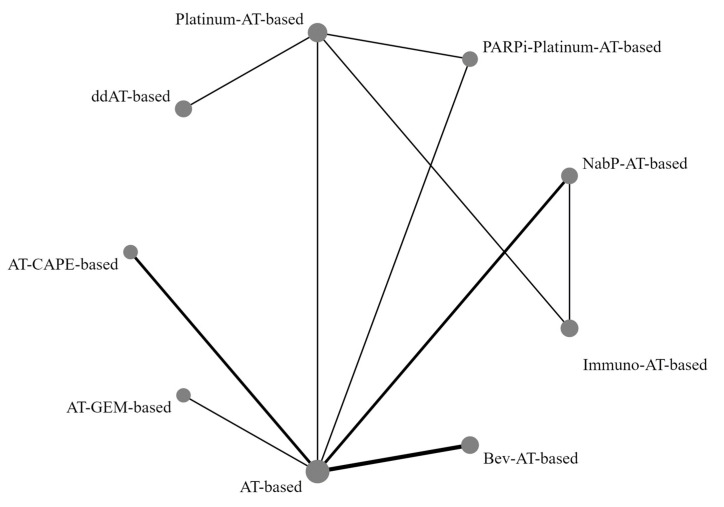
Network structure diagrams for pathologic complete response among different direct comparisons (using network meta-analysis, we can estimate the relative effectiveness of treatments when a direct comparison is not available). Direct comparisons are represented by the black lines connecting the neoadjuvant therapy regimens. Line width is proportional to the number of trials, including every pair of neoadjuvant regimens, whereas circle size is proportional to the total number of trials for each neoadjuvant regimen in the network. AT-based, anthracyclines/taxanes chemotherapy; ddAT, dose dense-anthracyclines/taxanes chemotherapy; AT-CAPE, anthracyclines/taxanes + capecitabine chemotherapy; AT-GEM, anthracyclines/taxanes + gemcitabine chemotherapy; Bev-AT, anthracyclines/taxanes + bevacizumab chemotherapy; Immuno-AT, anthracyclines/taxanes + immunotherapy-based chemotherapy; Nab-P-AT, anthracyclines + nab-Paclitaxel chemotherapy; PARPi-Platinum-AT, anthracyclines/taxanes + platinum agents + PARP inhibitors-based chemotherapy; Platinum-AT, anthracyclines/taxanes + platinum agents chemotherapy.

**Figure 3:**
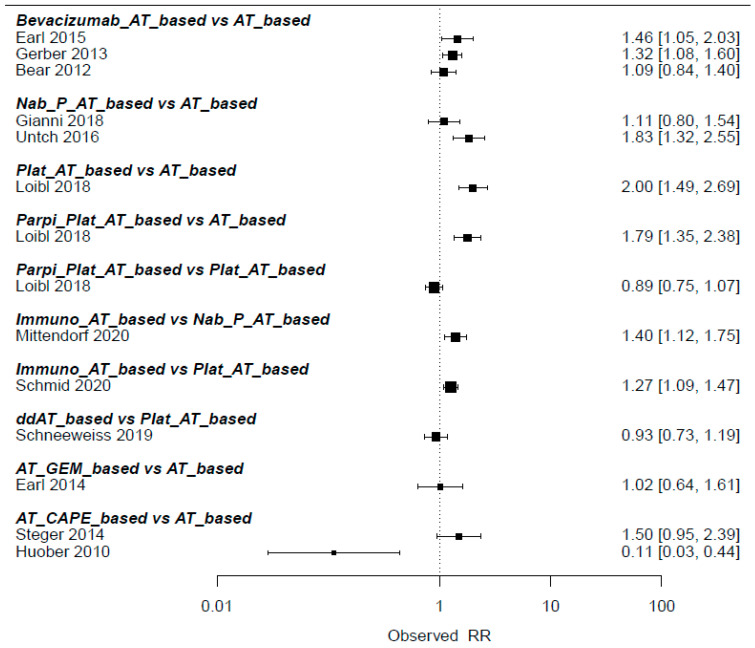
Pairwise direct comparisons of standard vs. more intense anthracycline/taxanes-based chemotherapy schedules (on the left, direct comparisons and authors/years of publication), on the right, the relative risk of pathologic complete response with experimental compared with control arms. AT-based, anthracyclines/taxanes chemotherapy [7,8,10,11,12,14,16,17,18]; ddAT, dose dense-anthracyclines/taxanes chemotherapy [13,15]; AT-CAPE, anthracyclines/taxanes + capecitabine chemotherapy [7,18]; AT-GEM, anthracyclines/taxanes + gemcitabine chemotherapy [10]; Bev-AT, anthracyclines/taxanes + bevacizumab chemotherapy [8,12,14]; Immuno-AT, anthracyclines/taxanes + immunotherapy-based chemotherapy [5,9]; Nab-P-AT, anthracyclines + nab-Paclitaxel chemotherapy [9,11,16]; PARPi-Platinum-AT, anthracyclines/taxanes + platinum agents + PARP inhibitors-based chemotherapy [17]; Platinum-AT, anthracyclines/taxanes + platinum agents chemotherapy [5,17].

**Figure 4:**
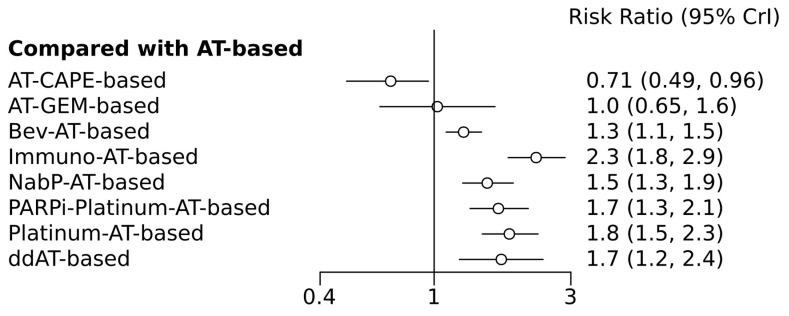
Forest plots of indirect network comparisons between different neoadjuvant chemotherapies (pathologic complete response) in breast cancer compared to the referent standard arm (anthracyclines/taxanes only chemotherapy). AT-based, anthracyclines/taxanes chemotherapy; ddAT, dose dense-anthracyclines/taxanes chemotherapy; AT-CAPE, anthracyclines/taxanes + capecitabine chemotherapy; AT-GEM, anthracyclines/taxanes + gemcitabine chemotherapy; Bev-AT, anthracyclines/taxanes + bevacizumab chemotherapy; Immuno-AT, anthracyclines/taxanes + immunotherapy-based chemotherapy; Nab-P-AT, anthracyclines + nab-Paclitaxel chemotherapy; PARPi-Platinum-AT, anthracyclines/taxanes + platinum agents + PARP inhibitors-based chemotherapy; Platinum-AT, anthracyclines/taxanes + platinum agents chemotherapy.

Network diagrams depicting various treatment comparisons in relation to pCR are presented in Figure 2, offering a visual representation of the complex interplay between different treatment modalities. Figure 3 shows the pairwise comparisons, giving a detailed account of direct treatment comparisons. The direct comparisons involved 10 combinations from 12 trials. Notably, Bayesian comparisons, detailed in Figure 4 and Supplementary Tables S1 and S2, revealed that nearly all regimens, with the notable exceptions of anthracycline/taxanes combined with gemcitabine or capecitabine, yielded a higher pCR than the standard regimen. This was evident in both direct and indirect comparison scenarios. Immunotherapy-based regimens, for instance, resulted in a pCR rate more than double that of historical regimens (relative risk [RR] = 2.3, 95% CI = 1.9–2.9), situating them as the top-ranked treatment with a staggering 99% probability of SUCRA effectiveness.

**Figure 5:**
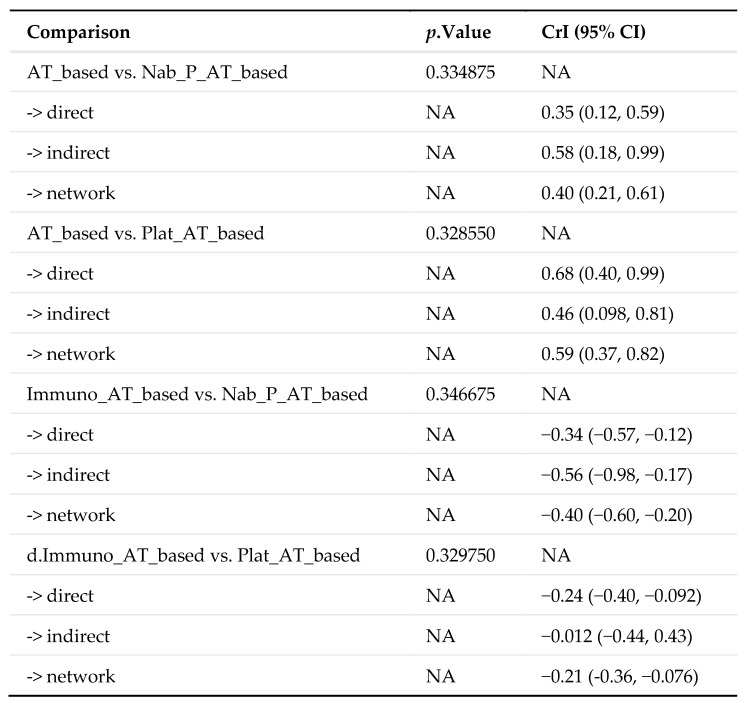
Local inconsistency assessment by the node-splitting analysis. AT-based, anthracyclines/taxanes chemotherapy; ddAT, dose dense-anthracyclines/taxanes chemotherapy; AT-CAPE, anthracyclines/taxanes + capecitabine chemotherapy; AT-GEM, anthracyclines/taxanes + gemcitabine chemotherapy; Bev-AT, anthracyclines/taxanes + bevacizumab chemotherapy; Immuno-AT, anthracyclines/taxanes + immunotherapy-based chemotherapy; Nab-P-AT, anthracyclines + nab-Paclitaxel chemotherapy; PARPi-Platinum-AT, anthracyclines/taxanes + platinum agents + PARP inhibitors-based chemotherapy; Platinum-AT, anthracyclines/taxanes + platinum agents chemotherapy.

The transitivity and consistency of the network studies for pCR were rigorously evaluated, as detailed in Figure 5. The analysis revealed that the consistency model was either similar or superior to the inconsistency model, affirming the presence of global consistency across studies. This was further bolstered by the results of the node-splitting analysis, which demonstrated no significant discrepancies between direct and indirect evidence, as per the Bayesian framework. This local consistency was a critical aspect of the study, ensuring the reliability of the findings. 

### 3.3. Disease-Free Survival 

**Figure 6:**
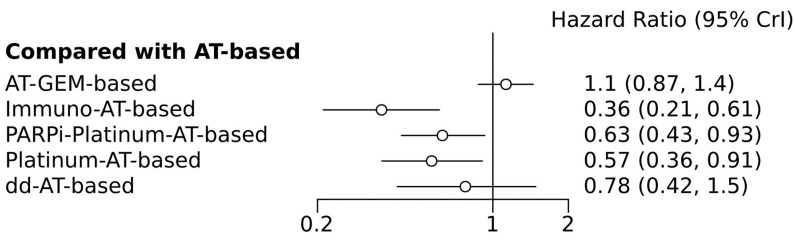
Forest plots of indirect comparisons between different neoadjuvant chemotherapies (disease-free survival/event-free survival) in breast cancer compared to the referent standard arm (anthracyclines/taxanes only chemotherapy). AT-based, anthracyclines/taxanes chemotherapy; ddAT, dose dense-anthracyclines/taxanes chemotherapy; AT-CAPE, anthracyclines/taxanes + capecitabine chemotherapy; AT-GEM, anthracyclines/taxanes + gemcitabine chemotherapy; Bev-AT, anthracyclines/taxanes + bevacizumab chemotherapy; Immuno-AT, anthracyclines/taxanes + immunotherapy-based chemotherapy; Nab-P-AT, anthracyclines + nab-Paclitaxel chemotherapy; PARPi-Platinum-AT, anthracyclines/taxanes + platinum agents + PARP inhibitors-based chemotherapy; Platinum-AT, anthracyclines/taxanes + platinum agents chemotherapy.

A network meta-analysis was conducted to evaluate DFS/EFS outcomes across five distinct treatment combinations. Direct comparisons involved five combinations from four trials. This analysis revealed that chemotherapy and immunotherapy significantly outperformed the standard neoadjuvant chemotherapy (including anthracycline and taxanes). The HR was 0.36 (95% CI = 0.21–0.61; Figure 6), highlighting the substantial improvement in DFS/EFS rates with immune checkpoint inhibitors. The evaluation of the effectiveness ranking of these treatments further underscored the superiority of the chemotherapy and immunotherapy combination. It achieved the highest P score of 0.95, indicating its preference among the options and an SUCRA probability of 1, confirming its status as the most efficacious choice. Conversely, chemotherapy based on platinum was shown to be less effective than regimens that included immune checkpoint inhibitors, with a hazard ratio of 1.58 (95% CI = 1.21–2.09). 

**Figure 7:**
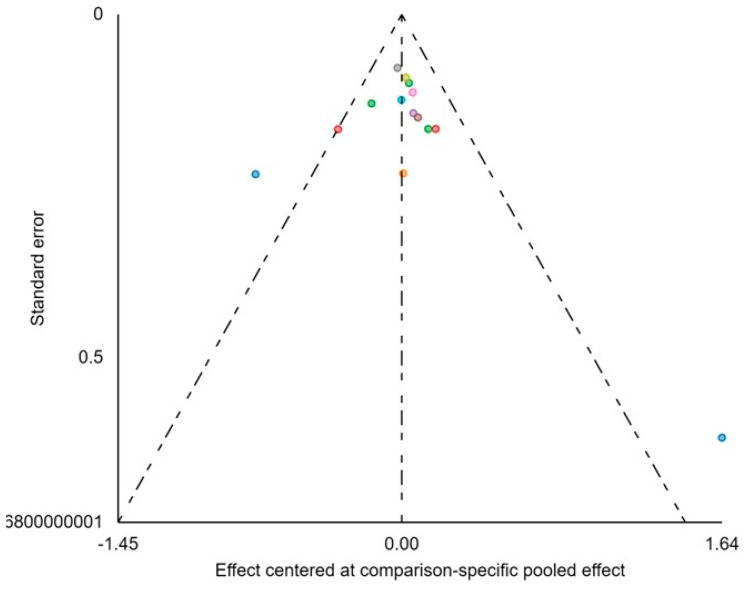
Adjusted funnel plots of all included publications on pCR.

Additionally, Figure 7 reports a funnel plot, providing a visual representation of the data’s distribution and aiding in the assessment of potential publication bias.

## 4. Discussion

Most patients with early-stage TNBC are now treated with neoadjuvant chemotherapy. This approach, primarily driven by the significant prognostic benefit associated with achieving pCR, particularly in patients with more aggressive subtypes, has become the standard of care. The rationale behind this strategy hinges on the aggressive nature of TNBC and the lack of effective targeted hormonal therapies for other BC subtypes. Neoadjuvant chemotherapy allows for the early assessment of treatment response, which can inform subsequent therapeutic decisions and potentially lead to better outcomes.

Over the past few years, significant research has been dedicated to determining the most effective chemotherapy regimen for enhancing the efficacy of neoadjuvant therapy. Network meta-analysis emerged as a critical tool in this research because most regimens have not been evaluated in direct head-to-head comparisons. For instance, there is a lack of direct comparative data between platinum-based or dose-dense schedules and newer treatments such as immune checkpoint inhibitors. This analysis is the most comprehensive and up-to-date NMA comparing the efficacy of all phase III neoadjuvant trials, including those investigating the addition of novel agents, such as immunotherapy, VEGF inhibitors, PARP inhibitors, and others, to standard chemotherapy.

The outcomes of this NMA suggest potential for improvement beyond the reference regimens of anthracycline plus taxane-based chemotherapy. These findings encapsulate and build upon all research conducted to date. Specifically, the integration of immunotherapy with conventional chemotherapy regimens has emerged as the most effective strategy. This approach was associated with a likelihood of achieving pCR more than twice as high as that of traditional chemotherapy alone. Furthermore, the use of immune checkpoint inhibitors resulted in a 64% reduction in the risk of disease progression or death compared with standard chemotherapy regimens.

These findings are consistent with the results from two recently published systematic reviews and network meta-analyses showing significant improvement in pCR rate and time-to-event outcomes associated with PD-1 inhibitor plus platinum and anthracycline- and taxane-based chemotherapy [20,21].

Triple-negative BC is a distinct and aggressive form of breast cancer, characterized by the absence of estrogen and progesterone receptors and lack of HER2 overexpression. This subtype is associated with poorer prognosis and limited treatment options compared to other breast cancer types. The challenge in treating TNBC stems from its heterogeneity and the absence of effective targeted therapies for hormone receptor- or HER2-positive breast cancers.

In recent years, neoadjuvant chemotherapy has become a cornerstone of the management of early-stage TNBC. This approach involves administering chemotherapy before surgical intervention. The primary goals of neoadjuvant chemotherapy are to reduce the tumor size, make it more amenable to surgery, and provide an early assessment of the tumor’s response to treatment. Achieving pCR after neoadjuvant chemotherapy is a key prognostic factor in TNBC, as patients who achieve pCR generally have a better overall prognosis. Traditional chemotherapy regimens for TNBC typically include anthracyclines and taxanes. However, the quest to improve outcomes for TNBC has led to the exploration of additional therapeutic agents and their combinations. Platinum-based chemotherapies, such as carboplatin and cisplatin, have shown promise in increasing the pCR rates in patients with TNBC. These agents are believed to be effective because of their ability to create interstrand DNA crosslinks, leading to apoptosis in cancer cells, particularly in those deficient in DNA repair mechanisms, such as BRCA-mutated tumors, which are more prevalent in TNBC.

Immunotherapy has emerged as a game-changer for the treatment of several cancers, including TNBC. Immune checkpoint inhibitors, such as pembrolizumab and atezolizumab, have shown significant efficacy when combined with chemotherapy for TNBC [19,22]. These drugs function by blocking PD-1/PD-L1, which enhances the immune system’s ability to recognize and attack cancer cells. Another area of active research on TNBC treatment involves the use of PARP inhibitors. These agents are particularly effective in patients with BRCA mutations, which are common in TNBC patients. PARP inhibitors function by exploiting the concept of synthetic lethality, targeting DNA repair pathways in cancer cells that are already compromised due to BRCA mutations. Furthermore, angiogenesis inhibitors such as bevacizumab have been explored in TNBC, although their role remains controversial. These agents target the VEGF pathway to inhibit tumor angiogenesis, thereby starving the tumor from its blood supply.

In a meta-analysis and NMA of immune checkpoint inhibitor studies in TNBC, despite a non-significant improvement in OS (except in one study), all drugs showed an invariably extended EFS/DFS, reducing the risk of progression or death and nearly doubling the control arm in pCR [23]. This benefit is offset by an increase in immune-related adverse events, such as hyperthyroidism, hypothyroidism, pneumonitis, and adrenal insufficiency, although atezolizumab was found to be safer than pembrolizumab. This implies that PD-1/PD-L1 inhibitors may be the most effective when started early in the disease course.

The findings of our study align with those of the pivotal phase III KEYNOTE-522 trial, which was a landmark study in this field [5]. The KEYNOTE-522 trial was the first to demonstrate that the addition of pembrolizumab, an immune checkpoint inhibitor, to neoadjuvant chemotherapy led to a statistically significant and clinically meaningful improvement in EFS in patients with early-stage TNBC. This study is crucial in illustrating the potential of immunotherapy to change the treatment landscape for this aggressive BC subtype. Beyond the studies included, other agents targeting the PD-1/PD-L1 axis were tested in the neoadjuvant setting in TNBC. In the GeparNuevo phase II study, patients with cT1b-cT4a-d TNBC received durvalumab or placebo in combination with nab-paclitaxel-based chemotherapy followed by surgery. Durvalumab was not continued postoperatively. Despite a non-significant increase in the pCR rate, significant differences were observed for 3-year invasive disease-free survival (iDFS), distant disease-free survival (DDFS), and OS [24].

Despite these advancements, challenges remain in terms of treatment. The heterogeneity of the disease means that not all patients respond to the same treatments. This underscores the need for personalized medical approaches, which could include genomic and molecular profiling of tumors, to identify specific vulnerabilities that can be targeted with tailored therapies. Additionally, while pCR is a useful early endpoint in clinical trials, it is not a perfect surrogate for long-term outcomes such as overall survival. Therefore, ongoing research and follow-up are crucial to better understand the long-term benefits of these emerging therapies. Moreover, the toxicity profiles of these treatments should be carefully considered. The combination of chemotherapy with novel agents can increase the risk of adverse effects, which must be weighed against the potential benefits. Patient selection and the management of side effects are critical components of treatment strategies.

It is also essential to acknowledge the limitations of this study. First, our research focused on an extensive review of significant phase III trials, deliberately excluding smaller phase 2 studies to reduce the risk of heterogeneity and bias. Second, there was uncertainty in our findings due to the variability in patient groups, treatment lengths, and medication dosages among the studies we considered. To address this, we employed strict criteria for selecting studies and meticulously evaluated the transitivity assumption. Third, the limited number of trials for some drug combinations weakened the reliability of the comparisons. Fourth, the small patient count in some comparisons led to broad 95% CrI effect sizes, increasing the risk of publication bias. Fifth, our study was primarily designed to assess the categories of neoadjuvant therapies, offering less insight into treatment timing, sequences, and dosage forms. Sixth, our study lacked access to individual patient data, hindering our ability to identify patients who could benefit from less intensive treatment. Seventh, the inconsistent availability of time-to-event data for neoadjuvant therapies restricts our ability to link treatment plans with survival advantages. Lastly, slight variations in how different trials defined DFS and EFS and our combined analysis of these outcomes might have introduced additional heterogeneity and potential bias. 

The decision to continue the immune checkpoint inhibitor (and its contribution) in the adjuvant setting depends on physician choice and patient tolerance to the primary treatment. Usually, when pembrolizumab is incorporated into neoadjuvant chemotherapy, the typically used regimen is the standard of care, according to the KEYNOTE-522 trial. Currently, no data are available regarding other regimens following neoadjuvant pembrolizumab treatment. It remains unclear whether the specific chemotherapy regimen is crucial to achieving the potential benefits of adding pembrolizumab or whether omitting carboplatin would affect long-term outcomes. Furthermore, the outcomes of using only adjuvant pembrolizumab or anthracycline-free regimen are unknown. Generally, pembrolizumab should be continued in the adjuvant phase regardless of whether pCR is achieved. The final publication of the outcome data is eagerly anticipated.

Another area of investigation involves the combination of PARP and immune checkpoint inhibitors. The rationale for this combination stems from their complementary actions. PARP inhibitors can increase the immunogenicity of cancer cells by inducing DNA damage and increasing neoantigen expression. This may make cancer cells more recognizable to the immune system. Furthermore, DNA damage inflicted by PARP inhibitors can trigger an inflammatory response, potentially making the tumor microenvironment more amenable to immune attack. Currently, various clinical trials are being conducted to explore these combinations.

The field of cancer treatment is expected to evolve rapidly. The integration of genomic and molecular profiling into clinical practice has led to the development of personalized and targeted therapies. Such advancements could allow oncologists to tailor treatment regimens to individual patients more precisely, potentially enhancing outcomes and reducing the risk of unnecessary toxicity.

In conclusion, the trials featured in the meta-analysis explored a variety of treatment combinations and covered different enrollment years. The possibility of selection bias also exists, as the studies primarily included early-stage TNBC patients who were carefully chosen and enrolled in high-quality trials at academic centers, all of whom had good performance status. In contrast, real-world cases might involve treating a broader range of patients, including the elderly, those with comorbidities, and those experiencing significant polypharmacy, who may not be suitable candidates for intensive multi-agent therapies. Furthermore, the primary endpoint chosen (pCR) is a useful early measure of treatment effectiveness, but it is not universally recognized as a definitive marker of long-term survival in BC. Additionally, these often lacked final survival data, and the awaited final publications could potentially revise present results.

However, our meta-analysis contributes significant evidence to the growing body of knowledge on optimizing treatment strategies for early-stage TNBC. This finding confirms that combining immunotherapy with neoadjuvant platinum-based chemotherapy is currently the most effective strategy for achieving significant pathological downstaging and improving clinical outcomes. However, the field of TNBC treatment is dynamic, and ongoing research is vital to further refine these strategies and explore new therapeutic avenues.

## Figures and Tables

**Table 1:** Characteristics of the included studies.

Author/Year	Type of Study	N° pts/Median Follow Up (Months)	Stage	Definition of TN Status	Treatment Arms (ctr vs. exp)	pCR % (ctr vs. exp)	Risk of Bias
Bear/2012	Prospective RCT	244/-	I–III	NA	D × 4 → AC × 4 DX × 4 → AC × 4 DG × 4 → AC × 4 ±Bevacizumab	47.1 vs. 51.2	Low
Earl/2014	Prospective RCT	157/47	I–III	ER/PR NA; HER2 = -, 1+, 2+/ISH-	EC × 4 → PAC × 4 PAC × 4 → EC × 4 EC × 4 → TG × 4 TG × 4 → EC × 4	31.5 vs. 32.1	Low
Earl/2015	Prospective RCT	241/-	I–III	ER/PR score = 0–2/8; HER2 = -, 1+, 2+/ISH-	D × 3 → FEC × 3 ±Bevacizumab	31.1 vs. 45.3	Low
Gerber/2013	Prospective RCT	663/-	I–III	ER/PR < 10%; HER2 = -, 1+, 2+/ISH-	EC × 4 → D × 4 ±Bevacizumab	32.9 vs. 43.3	Low
Gianni/2018	Prospective RCT	219/-	I–III	ER/PR < 1%; HER2 = -, 1+, 2+/ISH-	PAC × 4 → A regimen nabPAC × 4 → A regimen	37.2 vs. 41.2	Low
Huober/2010	Prospective RCT	89/-	I–III	ER/PR < 10%; HER2 = -, 1+, 2+/ISH-	TAC × 2 → TAC × 4-6 TAC × 2 → NX × 4	43.3 vs. 4.8	Moderate
Loibl/2018 and Geyer/2022	Prospective RCT	634/-	I–III	ER/PR < 1%; HER2 = -, 1+, 2+/ISH-	PAC + Carbo × 4 + Veliparib → AC × 4 PAC + Carbo × 4 + placebo → AC × 4 PAC + placebo → AC × 4	58.8 vs. 52.5	Low
Mittendorf/2020	Prospective RCT	333/20.2	II–III	NA	nabPAC × 12w → AC q14 × 4 ±Atezolizumab	41.0 vs. 57.5	Low
Schmid/2020 and Schmid/2022	Prospective RCT	602/15.5	II–III	ASCO guidelines	PAC + Carbo × 4 ± Pembrolizumab → AC/EC × 4 ± Pembrolizumab	51.2 vs. 64.8	Low
Schneeweiss/2019 and Schneeweiss/2022	Prospective	403/-	I–III	ER/PR < 1%; HER2 = -, 1+, 2+/ISH-	iddEPC PAC + M + Carbo × 18w	20.6 vs. 22.1	Moderate
Steger/2014	Prospective RCT	127/-	I–III *	ER/PR < 10%; HER2 = -, 1+, 2+/ISH-	ED × 6 EDX × 6	30.1 vs. 45.3	Moderate
Untch/2016	Prospective RCT	276/-	I–III	ER/PR < 1%; HER2 = -, 1+, 2+/ISH-	nabPAC × 4 → EC PAC × 4 → EC	26.2 vs. 48.2	Low

TN, triple-negative; pCR, pathological complete response; RCT, randomized controlled trial; ER, estrogen receptors; PR, progesterone receptors; HER2, human epidermal growth factor receptor 2; ISH, in situ hybridization; D, docetaxel; DX, docetaxel + capecitabine; DG, docetaxel + gemcitabine; AC, doxorubicin + cyclophosphamide; EC, epirubicin + cyclophosphamide; PAC, paclitaxel; TG, paclitaxel + gemcitabine; FEC, fluorouracil + epirubicin + cyclophosphamide; A, anthracycline chemo regimen; nabP, nab-paclitaxel; TAC, docetaxel + doxorubicin + cyclophosphamide; NX, vinorelbine + capecitabine; Carbo, carboplatin; M, nonpegylated liposomal doxorubicin; iddEPC, intense dose-dense epirubicin + paclitaxel + cyclophosphamide; ED, epirubicin + docetaxel; EDX, epirubicin + docetaxel + capecitabine; NA, not available; *, except T4d; PAC, paclitaxel.

## Data Availability

At request to the corresponding author.

## Supplementary Materials

The following supporting information can be downloaded at: https://www.mdpi.com/article/10.3390/medicina60020341/s1, Table S1: Comparison of the included interventions: risk ratio (95% CrI); Table S2: Rank probabilities table (the rank probability of the 9 regimens for pCR.
