# Supplementary material for: Different Chemotherapy Regimens and Pathologic Complete Response in Triple-Negative Breast Cancer: An Updated Network Meta-Analysis of Phase 3 Trials
Source: Medicina (Kaunas). 2024 Feb 19;60(2):341. doi: 10.3390/medicina60020341 (PMC10890456; doi:10.3390/medicina60020341)
Supplement: Supplementary file 1 [file medicina-60-00341-s001.zip › medicina-2803096-supplementary.pdf]

Supplementary Table S1. Comparison of the included interventions: risk ratio (95% CrI). Each cell gives the effect of the column-defining intervention relative to the row-defining intervention. Risk ratio > 1 means better pCR rates for treatments in the columns compared to treatments in the rows, and < 1 means worst pCR rates for ~~treaments~~treatments in the columns compared to treatments in the rows. Grey ~~colour~~color represent the direct comparisons. Bold character highlights the significant differences.

**AT-CAPE-based**	1.46 (0.83, 2.60)	**1.41 (1.04, 2.02)**	**1.80 (1.29, 2.61)**	**3.23 (2.20, 4.89)**	**2.18 (1.51, 3.28)**	**2.39 (1.63, 3.66)**	**2.60 (1.79, 3.96)**	**2.44 (1.54, 3.93)**
	**AT-GEM-based**	0.97 (0.61, 1.54)	1.23 (0.75, 2.00)	**2.22 (1.30, 3.70)**	1.49 (0.90, 2.47)	1.63 (0.97, 2.74)	1.78 (1.06, 2.99)	1.66 (0.94, 2.94)
		**AT-based**	**1.26 (1.10, 1.46)**	**2.27 (1.81, 2.86)**	**1.53 (1.25, 1.88)**	**1.67 (1.33, 2.13)**	**1.83 (1.47, 2.30)**	**1.71 (1.22, 2.39)**
			**Bev-AT-based**	**1.79 (1.37, 2.34)**	1.20 (0.94, 1.54)	**1.32 (1.01, 1.74)**	**1.44 (1.11, 1.88)**	1.35 (0.94, 1.93)
				**Immuno-AT-based**	**0.67 (0.55, 0.81)**	**0.73 (0.59, 0.92)**	**0.80 (0.69, 0.92)**	0.75 (0.57, 1.00)
					**NabP-AT-based**	1.09 (0.85, 1.41)	1.19 (0.96, 1.49)	1.11 (0.80, 1.54)
						**PARPi-Platinum-AT-based**	1.09 (0.90, 1.30)	1.02 (0.74, 1.38)
							**Platinum-AT-based**	0.93 (0.73, 1.19)
								**ddAT-based**

AT-based, anthracyclines/taxanes chemotherapy; ddAT, dose dense-anthracyclines/taxanes chemotherapy; AT-CAPE, anthracyclines/taxanes + capecitabine chemotherapy; AT-GEM, anthracyclines/taxanes + gemcitabine chemotherapy; Bev-AT, anthracyclines/taxanes + bevacizumab chemotherapy; Immuno-AT, anthracyclines/taxanes + immunotherapy-based chemotherapy; Nab-P-AT, anthracyclines + nab-Paclitaxel chemotherapy; PARPi-Platinum-AT, anthracyclines/taxanes + platinum agents + PARP inhibitors-based chemotherapy; Platinum-AT, anthracyclines/taxanes + platinum agents chemotherapy.

Supplementary Table S2. Rank probabilities table (the rank probability of the 9 regimens for pCR. Rank 1 represents the best treatment and rank 9 represents the worst.

	Rank 1	Rank 2	Rank 3	Rank 4	Rank 5	Rank 6	Rank 7	Rank 8	Rank 9
**AT-CAPE-based**	0	0	0	0	0	0	0	0.09	0.90
**AT-GEM-based**	0	0.01	0	0.01	0.03	0.13	0.33	0.36	0.09
**AT-based**	0	0	0	0	0	0	0.45	0.54	0
**Bev-AT-based**	0	0	0	0.01	0.08	0.71	0.18	0	0
**Immuno-AT-based**	0.97	0.02	0	0	0	0	0	0	0
**NabP-AT-based**	0	0.03	0.08	0.24	0.54	0.08	0	0	0
**PARPi-Platinum-AT-based**	0	0.13	0.26	0.40	0.16	0.02	0	0	0
**Platinum-AT-based**	0	0.54	0.38	0.06	0	0	0	0	0
**ddAT-based**	0.02	0.24	0.25	0.25	0.16	0.04	0	0	0

AT-based, anthracyclines/taxanes chemotherapy; ddAT, dose dense-anthracyclines/taxanes chemotherapy; AT-CAPE, anthracyclines/taxanes + capecitabine chemotherapy; AT-GEM, anthracyclines/taxanes + gemcitabine chemotherapy; Bev-AT, anthracyclines/taxanes + bevacizumab chemotherapy; Immuno-AT, anthracyclines/taxanes + immunotherapy-based chemotherapy; Nab-P-AT, anthracyclines + nab-Paclitaxel chemotherapy; PARPi-Platinum-AT,

anthracyclines/taxanes + platinum agents + PARP inhibitors-based chemotherapy; Platinum-AT, anthracyclines/taxanes + platinum agents chemotherapy.
